# Molecular Dynamics Study of Functionalized Gold Nanoparticles: Structural and Aggregation Behavior under Varying Ionic Strength

**DOI:** 10.1021/acsphyschemau.5c00077

**Published:** 2025-10-17

**Authors:** Esequias Coelho, Douglas Xavier de Andrade, Agnaldo Rosa de Almeida, Guilherme Colherinhas

**Affiliations:** † Instituto de Física, 67824 Universidade Federal de Goiás, Goiânia, GO 74690-900, Brazil; ‡ 28095 Instituto Federal de Educação, Ciência E Tecnologia de Goiás, Aparecida de Goiânia, GO 74968-755, Brazil; § CampusAnápolis de Ciências Exatas e Tecnológicas, 271384 Universidade Estadual de Goiás, Anápolis, GO 75.132-400, Brazil

**Keywords:** molecular dynamics, gold nanoparticle, ionic interaction, H-bond, H-bond lifetime

## Abstract

This study employed classical molecular dynamics simulations to explore the interactions between functionalized gold nanoparticles (AuNPs) and monovalent ions in aqueous environments. Two AuNP models were analyzed: type I, positively charged (Au_144_(SRNH_3_^+^)_60_), and type II, negatively charged (Au_144_(SRCOO^–^)_60_), both functionalized with 60 organic thiolate ligands (SR). Four systems were constructed to examine the effects of ionic strength and nanoparticle concentration: (i) one AuNP of each type in pure water; (ii) the same system with 60 Na^+^ and 60 Cl^–^ ions; (iii) two AuNPs of each type in pure water; and (iv) the same configuration as (iii), with 120 Na^+^ and 120 Cl^–^ ions. Simulations focused on interparticle interactions, hydrogen-bonding dynamics, and the roles of electrostatic and van der Waals forces. Results show that ionic strength and nanoparticle concentration significantly affect the system’s energy distribution and structural organization. Ionic screening reduces electrostatic interactions, modifies hydrogen bond lifetimes, and induces the rearrangement of hydration shells around the nanoparticles. Additionally, variations in ion distribution impact the spatial organization and mobility of solvated species. These findings provide molecular-level insights into ion-mediated nanoparticle interactions and are crucial for the rational design of functional nanomaterials in biomedical, catalytic, and materials science applications.

## Graphical abstract

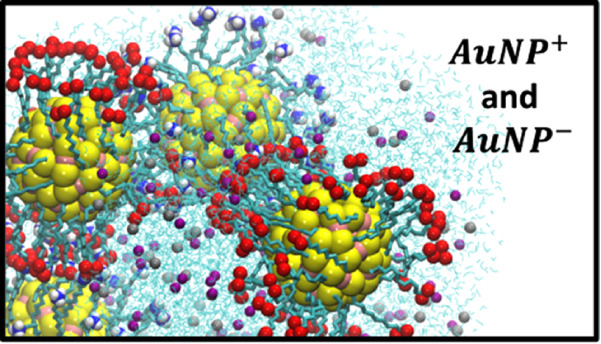

## 1 Introduction

The investigation of nanoparticle (NP) interactions within biological environments has garnered considerable attention in recent years, owing to their vast potential in nanomedicine, biotechnology, and materials science fields.^1–5^ Upon exposure to biological fluids, nanoparticles undergo a competitive adsorption process involving various biomolecules—a phenomenon known as protein corona formation—which plays a central role in determining their fate, functionality, and biocompatibility.^6^ Gold nanoparticles (AuNPs), in particular, are distinguished by their unique combination of physicochemical properties, including high chemical stability, tunable surface functionalization, and excellent biocompatibility. These characteristics make them highly promising platforms for applications such as targeted drug delivery, bioimaging, biosensing, and photothermal therapy.^7–10^ In addition to biomedical applications, AuNPs are extensively employed in fields like optoelectronics, catalysis, and cosmetology.^11–20^ In cosmetic formulations, they have been incorporated into antiaging creams, antimicrobial products, and wound-healing applications due to their antimicrobial properties and influence on cell regeneration processes.^13,14^ These functionalities are closely associated with their structural and optical characteristics, including size-dependent surface plasmon resonance and fluorescence behavior.^21–29^

Structurally, AuNPs can be described by the general formula (Au)*_n_* (SR)*_m_*, in which a gold core is stabilized by thiolate ligands covalently bonded through gold–sulfur interactions. Common stoichiometries include Au_12_(SR)_18_, Au_38_(SR)_24_), and Au_144_(*SR*)_60_ models.^30–35^ The ligand shell not only imparts colloidal stability but also provides a versatile platform for further functionalization with biomolecules such as peptides, nucleic acids, or antibodies.^36–42^ The ability to exert precise molecular control over both core size and ligand composition has facilitated the applications of these particles in a wide range of fields, including catalysis,^43,44^ biosensing, and chemiresistive devices.^45^ Ultrasmall AuNPs (diameter <2 nm) with defined atomic structures and narrow size distributions offer additional advantages for applications requiring reproducible and predictable physicochemical behavior.^35^ However, while considerable attention has been devoted to the interactions between AuNPs and biomolecules, their behavior in aqueous ionic environments—particularly their interactions with monovalent and divalent ions—remains underexplored. A comprehensive understanding of how ionic composition influences the solvation, stability, and aggregation of AuNPs is essential for optimizing their performance in both biological and environmental systems.

Recent studies have highlighted the critical role of long-range electrostatic interactions—modulated by the surrounding aqueous medium—in shaping the structural and dynamic properties of charged AuNPs.^46,47^ Using atomistic simulations, Heikkilä et al.^47^ investigated the behavior of Au_144_(SR)_60_ nanoparticles in water and reported that solvent-mediated interactions are highly dependent on the nanoparticle’s surface charge and ligand chemistry. In a subsequent study, the same research group demonstrated that anionic AuNPs interact asymmetrically with lipid membranes, further emphasizing the role of electrostatic potential in governing both aggregation behavior and membrane affinity.^46^ More recently, G. Bordoni and G. Colherinhas^48^ have employed molecular dynamics simulations to investigate the aggregation behavior of negatively charged AuNPs [Au_144_(SC_11_H_22_COO^–^)_60_] in aqueous Na^+^ solutions. Their findings revealed that increasing NP concentration promotes clustering, primarily driven by nanoparticle–water interactions. Similarly, Coelho et al.^49^ examined the impact of different ionic compositions on the dynamics of anionic AuNPs, demonstrating that divalent ions (e.g., Ca^2+^, Mg^2+^) exhibit stronger interactions with NP surfaces than monovalent ions (e.g., Na^+^, K^+^), ultimately influencing hydration structure and NP mobility. Despite these advances, a comprehensive understanding of how positively and negatively charged AuNPs behave under varying ionic strengths remains limited. In this context, classical molecular dynamics (MD) simulations offer a robust and accurate framework for probing the energetic, structural, and dynamic characteristics of these systems in silico.^50–59^ These computational approaches enable a detailed analysis of hydrogen-bonding patterns, ionic distributions, solvation dynamics, and interparticle interactions, providing atomistic-level insights that complement experimental observations.

The present study aims to investigate the behavior of functionalized AuNPs bearing either positive $\left(-NH_{3}^{+}\right)$ or negative (−COO^–^) surface charges in aqueous media containing sodium and chloride ions, using MD simulations. Four distinct systems were constructed by varying both the number of AuNPs and the ionic concentrations. The analyses focused on interparticle distances, hydrogen-bonding dynamics, the spatial organization of water molecules and ions, and interaction energies—including Coulomb (*E_C_*) and Lennard–Jones (*E_LJ_*) interaction energy. By exploring how ionic strength and NP concentration modulate these properties, this work provides valuable insights into the fundamental principles governing ion–AuNP interactions in solution, thereby contributing to the rational design of functional nanomaterials for biomedical and technological applications.

## 2 Methodology

Classical MD simulations were employed to investigate the interactions between functionalized AuNPs in aqueous solution under varying charge states and ionic strengths. Two types of AuNPs were considered: type-I (positively charged) and type-II (negatively charged), both comprising a 144-atom gold core (Au_144_) functionalized with 60 organic ligands (SR), as illustrated in Figure 1. These ligands belong to the general class of organic thiolates (SR), where sulfur (*S*) forms a covalent bond with the gold surface and *R* denotes an alkyl chain. The *R* group employed in this study was C_11_H_22_, covalently attached to the Au_144_ core via a sulfur atom. Although the ligands themselves are neutral, the overall charge of the NP is determined by the terminal functional group attached to the *R* chain. The incorporation of terminal $-NH_{3}^{+}$ groups resulted in positively charged nanoparticles, whereas the presence of −COO^–^ groups imparted a negative charge. Specifically, the type-I AuNPs were capped with protonated amine terminal groups $\left(-NH_{3}^{+}\right)$ , yielding $Au_{144}{\left(SRNH_{3}^{+}\right)}_{60}$ , while type-II AuNPs were terminated with carboxylate groups (−COO^–^), resulting Au_144_(SRCOO^–^)_60_. Consequently, each NP carried a net atomic charge of ±60*e*, corresponding to the number of charged terminal groups. To reduce computational expense, a united-atom representation was adopted, wherein each methylene group (−CH_2_−) was modeled as a single interaction site. Molecular structures were constructed in accordance with the protocol outlined by Heikkilä et al.^47^ Interatomic interactions were parametrized using the OPLS-AA force field,^60^ which is widely recognized for its applicability to organic and biomolecular systems. Water molecules were represented using the Simple Point Charge (SPC) model,^61^ while sodium (Na^+^) and chloride (Cl^–^) ions were both parametrized with the OPLS-AA force field.^60^ Importantly, no modifications to the original force field parameters or to the covalent bonding description of Au–S and ligand topologies were introduced, ensuring full reproducibility and methodological consistency with the reference model.

**Figure 1:**
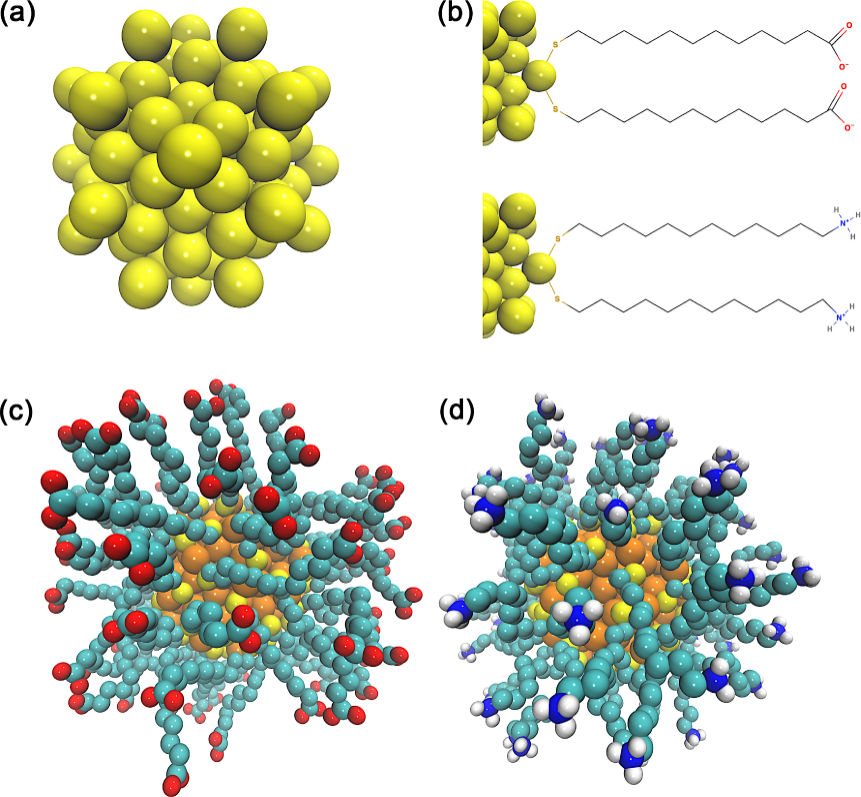
(a) Gold core of the AuNP. (b) Schematic representation of negatively and positively functionalized AuNP structures. (c) Negatively functionalized AuNP. (d) Positively functionalized AuNP. Atom color code: yellow = gold; orange = sulfur; cyan = carbon; red = oxygen; dark blue = nitrogen; white = hydrogen. The bonding scheme is shown in a simplified manner for visual clarity and does not represent modifications to the original molecular topology or force field parameters used in the simulations.

Four molecular systems were designed to explore the effects of NPs concentration and ionic strength. In system 1, one type I (positively charged) and one type II (negatively charged) AuNP were placed in pure water, with no added ions, and system 2 retained the same NP configuration, but 60 sodium (Na^+^) and 60 chloride (Cl^–^) ions were added to simulate moderate ionic strength. System 3 comprised two AuNPs of each type (two type I and two type II) in pure water molecules, and system 4 featured the same NP configuration as system 3, along with 120 Na^+^ and 120 Cl^–^ ions, representing a higher ionic strength environment. All systems were constructed using Packmol^62^ and placed in cubic simulation boxes with an initial volume of 343 nm^3^. A detailed overview of system composition—including the total number of atoms, average final volume, and AuNP molar concentration (mol·mL^– 1^)—is presented in Table 1.

**Table 1:** Composition of the Simulation Boxes Containing AuNP, Ions, and Water Molecules^a^

	#AuNPs Pos:Neg	#Ions Cl^–^:Na^+^	# Water	Initial Volume	Final Volume	*M* (10^‑5^mol/mL)
System 1	1:1	0:0	29970	343.0	350.0 ± 0.9	0.95
System 2	1:1	60:60	29586	343.0	347.3 ± 0.9	0.96
System 3	2:2	0:0	26136	343.0	354.1 ± 0.9	1.87
System 4	2:2	120:120	25545	343.0	352.7 ± 0.8	1.88

a The initial volume refers to the exact volume (in nm^3^) of the system as generated using the Packmol program, while the final volume corresponds to the average volume (in nm^3^ ± RMSD), calculated over the last 100 ns of the *MD* simulation. The molar concentration (*M*) of AuNPs in water is also reported (in mol/mL).

MD simulations were carried out using the GROMACS software package,^63^ under controlled temperature and pressure conditions, following a simulation protocol previously demonstrated to be effective for solvated organic systems.^58,64–68^ Each system initially underwent a 50 ns equilibration phase, starting with an NVT ensemble to stabilize the temperature, followed by an NPT ensemble to equilibrate the pressure and system density prior to production. Following equilibration, a 100 ns production run was conducted under only NPT conditions. Temperature was maintained at 300 K using the velocity-rescaling thermostat (v-rescale),^69^ while pressure was set at 1.013 bar using the isotropic Parrinello–Rahman barostat.^64^ The LINCS algorithm^70^ was applied to constrain covalent bonds involving hydrogen atoms, thereby enabling stable integration with a time step of 1 fs. During the production phase, 100 million configurations were generated, from which 50000 were sampled for subsequent statistical analysis. Long-range electrostatic interactions were treated using the particle-mesh Ewald (PME) method^71^ with a real-space cutoff of 1.2 nm, while Lennard–Jones (van der Waals) interactions were computed using a standard cutoff scheme with the same distance threshold of 1.2 nm.

Hydrogen bonding (HB) dynamics were analyzed using the methodology proposed by Luzar, Chandler, and van der Spoel,^72–74^ which incorporates both geometric and temporal criteria. An HB was considered to exist when the distance between donor and acceptor atoms was ≤3.5 Å and the angle between acceptor, hydrogen, and donor was ≤30^°^. To assess the stability and lifetime of HBs, the HB’s autocorrelation function was calculated throughout the simulation. This analysis also enables the estimation of the Gibbs free energy associated with HB’s formation and dissociation by accounting for both spatial and angular distributions. This approach offers a detailed molecular-level perspective of how ionic strength and NP concentration modulate interparticle interactions and influence the structure and dynamics of the surrounding HB network in the aqueous environment. All molecular visualizations and structural representations were generated using the VMD software package.^75^

## 3 Results and Discussion

### 3.1 Coulomb Interaction Energy

To elucidate the role of electrostatic interactions in governing the organization and behavior of AuNPs in aqueous ionic environments, we calculated the average Coulomb interaction energies (*E_C_*) for different pairwise combinations among AuNPs, water molecules, and ions across four molecular systems (Tables 2 and 3). While Coulombic and Lennard–Jones interaction energies obtained from classical MD are not intended for direct quantitative comparison with experimental measurements, they provide robust comparative descriptors within a consistent force-field framework. The observed trends, validated by independent structural and dynamical analyses, reinforce the mechanistic interpretation of interparticle interactions and aggregation pathways.

**Table 2:** Average Coulomb Interaction Energies (*E_C_*) for the Interaction of AuNPs with Different Types of AuNPs, AuNPs–Water, and AuNPs–Ions (in kJ/mol, per Number of AuNP) across Systems 1–4^a^

Interaction	System 1	System 2	System 3	System 4
AuNP^+^–AuNP^+^	–10,195 ± 25	–10,472 ± 14	–10,107 ± 18	–10,396 ± 25
AuNP^–^–AuNP*^–^*	–10,447 ± 20	–10,573 ± 18	–10,262 ± 40	–10,554 ± 22
AuNP^+^–AuNP*^–^*	–13,852 ± 150	–12,204 ± 120	–15,657 ± 208	–12,701 ± 230
AuNP^+^–H_2_O	–6,298 ± 92	–6,822 ± 90	–4,733 ± 120	–6,517 ± 145
AuNP^–^–H_2_O	–13,516 ± 120	–14,624 ± 9	–12,331 ± 85	–14,038 ± 150
AuNP^+^–Cl^–^	—	–312 ± 4	—	–376 ± 8
AuNP^+^–Na^+^	—	50 ± 2	—	107 ± 10
AuNP^–^–Cl^–^	—	33 ± 2	—	74 ± 3
AuNP^–^–Na^+^	—	–443 ± 11	—	–718 ± 23

a RMSDs values shown for all average interaction.

**Table 3:** Average Coulomb Interaction Energies (*E*_*C*_) of Individual AuNPs (#1 and #2) Interaction (in kJ/mol) for Systems 1–4^a^

Interaction	System 1	System 2	System 3	System 4
$\mathrm{AuN}P_{\#1}^{+}-\mathrm{AuN}P_{\#1}^{+}$	–10,195 ± 25	–10,472 ± 14	–10,226 ± 25	–10,258 ± 35
$\mathrm{AuN}P_{\#1}^{+}-\mathrm{AuN}P_{\#2}^{+}$	—	—	140 ± 12	3 ± 2
$\mathrm{AuN}P_{\#2}^{+}-\mathrm{AuN}P_{\#2}^{+}$	—	—	–10,127 ± 17	–10,537 ± 13
$\mathrm{AuN}P_{\#1}^{-}-\mathrm{AuN}P_{\#1}^{-}$	–10,447 ± 20	–10,573 ± 18	–10,269 ± 35	–10,440 ± 26
$\mathrm{AuN}P_{\#1}^{-}-\mathrm{AuN}P_{\#2}^{-}$	—	—	108 ± 28	198 ± 8
$\mathrm{AuN}P_{\#2}^{-}-\mathrm{AuN}P_{\#2}^{-}$	—	—	–10,363 ± 17	–10,865 ± 10
$\mathrm{AuN}P_{\#1}^{+}-\mathrm{AuN}P_{\#1}^{-}$	–13,852 ± 150	–12,204 ± 120	–7,035 ± 160	–5,725 ± 130
$\mathrm{AuN}P_{\#1}^{+}-\mathrm{AuN}P_{\#2}^{-}$	—	—	–8,316 ± 62	–9,822 ± 180
$\mathrm{AuN}P_{\#2}^{+}-\mathrm{AuN}P_{\#1}^{-}$	—	—	–8,734 ± 110	–9,854 ± 150
$\mathrm{AuN}P_{\#2}^{+}-\mathrm{AuN}P_{\#2}^{-}$	—	—	–7,229 ± 83	–0.001 ± 0.0

a RMSDs are shown for all average interaction.

On average, the interaction energies between AuNPs of the same charge type (i.e., positive–positive and negative–negative) remained relatively stable across all simulated systems, exhibiting only minor fluctuations regardless of the presence of ions. Specifically, *E_C_* for positively charged AuNPs ranged from −10,107 to −10,472 kJ/mol, while those for negatively charged AuNPs ranged from −10,262 to −10,573 kJ/mol. These consistently strongly negative values indicate stable internal charge distributions and support the conclusion that the structural integrity of individual AuNPs is maintained under varying ionic strength conditions. In contrast, the interaction between oppositely charged AuNPs (i.e., AuNP^+^ *–*AuNP*^–^*) is significantly more intense, indicative of a stronger attraction. The most pronounced interaction was observed in system 3 (−15,657 kJ/mol), which contained four nanoparticles in pure water, thereby maximizing direct electrostatic interactions. However, this attractive interaction was notably reduced in the presence of ions, as observed in systems 2 and 4, with values of −12,204 and −12,701 kJ/mol per AuNP, respectively. This attenuation is attributed to the electrostatic screening effect exerted by surrounding Na^+^ and Cl^–^ ions, which mitigate the direct Coulomb attractive force between oppositely charged NP by redistributing local charge density in the solvent environment. AuNP–H_2_O interactions also confirmed the expected electrostatic behavior. Negatively charged AuNPs exhibited significantly stronger interactions with surrounding water molecules with *E_C_* ranging from −12,331 to −14,624 kJ/mol, compared to −4,733 to −6,822 kJ/mol, for positively charged AuNPs. This disparity is primarily attributed to the strong electrostatic attraction between the negatively charged carboxylate functional groups and the partial positive charges on water hydrogens. These results suggest that anionic AuNPs promote the formation of more structured solvation shells and exert a greater influence on the reorganization of the local HB’s network in aqueous environments. AuNP-ion interactions further underscore the role of electrostatic complementarity in modulating system behavior. AuNPs^+^ exhibit strong attraction to Cl^–^ ions with interaction energies ranging from −312 to −376 kJ/mol, while simultaneously repelling Na^+^ ions, as reflected by positive interaction energies ranging from +50 to +107 kJ/mol, per AuNP. Conversely, AuNPs^–^ demonstrated strong attraction to Na^+^ ions ranging from −443 to −718 kJ/mol and repulsion from Cl^–^ in order of +33 to +74 kJ/mol, per AuNP. These interactions play a crucial role in the formation of diffuse ionic layers—commonly referred to as electric double layers—around the nanoparticles surfaces, thereby influencing the effective surface charge and modulating interparticle electrostatic potentials in solution.

Table 3 presents a detailed decomposition of Coulomb interactions energy (*E_C_*) between specific pairs of AuNPs. This level of detail enables the identification of symmetry-breaking effects, positional heterogeneity, and local structural rearrangements within the simulation box. Self-interactions (e.g., $AuNP_{\#1}^{+}-AuNP_{\#1}^{+}$ ) remained strongly negative ranging from −10,195 to −10,472 kJ/mol, in agreement with the global average values reported in Table 2. These results reaffirm the internal electrostatic cohesion of individual nanoparticles, which remains stable irrespective of the ionic strength or system size. Interparticle interactions between like-charged (e.g., $AuNP_{\#1}^{+}-AuNP_{\#2}^{+}$ ) exhibit clear electrostatic repulsion, with an interaction energy of 140 kJ/mol observed in system 3 (absence of ions), which decreases considerably to 3.0 kJ/mol in system 4 (presence of ions). A comparable trend is observed for negative AuNP pairs, although it slightly increases with higher ionic concentration. These observations underscore the effectiveness of electrostatic screening by solvated ions in attenuating repulsive forces, thereby facilitating closer spatial arrangements among like-charged AuNPs in ionic environments. Interactions between oppositely charged nanoparticles (e.g., $AuNP_{\#1}^{+}-AuNP_{\#1}^{-}$ ) account for the most attractive Coulomb contributions in the system. In system 1 (absence of ions), this interaction reaches −13,852 kJ/mol, while in system 2 (presence of ions), it is attenuated to −12,204 kJ/mol, reflecting the screening effect of the ionic environment. In systems 3 and 4, which contain four AuNPs, the attractive interactions are distributed across multiple particle pairs and exhibit considerable variability ranging from −9,854 to only −0.001 kJ/mol. This broad range of energies suggests that the nanoparticles adopt distinct and relatively persistent configurations rather than dynamically sampling multiple states. The observed differences likely reflect long-lived spatial arrangements and limited configurational exchange over the simulation time scale. The near-zero interaction value observed in system 4 $AuNP_{\#2}^{+}-AuNP_{\#2}^{-}$ indicates either complete electrostatic shielding by surrounding ions or substantial interparticle separation, limiting direct interaction.

Collectively, the results presented in Tables 2 and 3 provide compelling evidence that ionic strength significantly plays a key role in modulating the electrostatic landscape of AuNP systems. In the absence of ions, oppositely charged nanoparticles exhibit strong pairwise attraction with spatial organization primarily governed by Coulomb forces. As the ionic concentration increases, these interactions are progressively attenuated due to electrostatic screening by the solvated ions, resulting in diminished interparticle forces and more randomized distributions. Additionally, water actively mediates NP interactions—particularly for negatively charged AuNPs—where enhanced solvation contributes substantially to overall system stabilization and local structural organization. The integration of detailed pairwise analyses with global electrostatic interaction trends underscores the pronounced sensitivity of AuNP systems to both their electrostatic context and spatial configuration. These findings provide critical insight into nanoparticle behavior under biologically relevant conditions since ionic strength and spatial arrangement significantly influence system dynamics. Such insights are instrumental for the rational design of nanomaterials in applications such as drug delivery, biosensing, and catalysis, where control over interparticle spacing and ionic environments is essential for optimizing functional performance.

### 3.2 van der Waals Interaction Energy

van der Waals interactions, represented by Lennard–Jones (*E_LJ_*) potential energies, play a key role in the structural organization and dynamic behavior of functionalized AuNPs, particularly in complementing electrostatic forces in aqueous media. Tables 4 and 5 present the average interaction energies *E_LJ_* for various pairwise combinations, including both intra- and intertype AuNP–AuNP, as well as interactions between AuNP–H_2_O and AuNP–ions interactions. These values are reported across four distinct simulation configurations, each differing in ionic strength and *NP* concentration, thereby enabling a comprehensive assessment of van der Waals contributions under biologically and technologically relevant conditions.

**Table 4:** Average Lennard–Jones Interaction Energies (*E_LJ_*) for Interaction of AuNPs with Different Types of AuNPs, AuNPs–Water, and AuNPs–Ions (in kJ/mol, per Number of AuNP) across Systems 1–4^a^

Interaction	System 1	System 2	System 3	System 4
AuNP^+^–AuNP^+^	–3,314.8 ± 2.7	–3,322.1 ± 6.7	–3,374.2 ± 9.9	–3,347.4 ± 8.0
AuNP^–^–AuNP^–^	–3,670.8 ± 3.9	–3,747.9 ± 4.3	–3,908.8 ± 9.9	–3,823.5 ± 6.9
AuNP^+^–AuNP^–^	440.3 ± 11.0	225.7 ± 5.8	553.9 ± 18.3	369.9 ± 10.6
AuNP^+^–H_2_O	–357.8 ± 17.0	–245.7 ± 4.4	–541.4 ± 13.0	–294.3 ± 12.0
AuNP^–^–H_2_O	–574.8 ± 10.0	–395.4 ± 4.4	–603.9 ± 7.5	–421.5 ± 13.2
AuNP^+^–Cl^–^	—	–20.5 ± 0.2	—	–25.7 ± 0.4
AuNP^+^–Na^+^	—	–0.3 ± 0.01	—	–0.7 ± 0.02
AuNP^–^–Cl^–^	—	–3.4 ± 0.2	—	–8.7 ± 0.4
AuNP^–^–Na^+^	—	–12.6 ± 0.1	—	–2.7 ± 0.1

a RMSDs are shown for all average interaction.

**Table 5:** Average Lennard–Jones Interaction Energies (*E_LJ_*) of Individual AuNPs Interaction (in kJ/mol) for Systems 1–4^a^

Interaction	System 1	System 2	System 3	System 4
$\mathrm{AuN}P_{\#1}^{+}-\mathrm{AuN}P_{\#1}^{+}$	–3,314.8 ± 2.7	–3,322.1 ± 6.7	–3,357.1 ± 8.3	–3,351.4 ± 9.8
$\mathrm{AuN}P_{\#1}^{+}-\mathrm{AuN}P_{\#2}^{+}$	—	—	–8.6 ± 0.5	–0.1 ± 0.07
$\mathrm{AuN}P_{\#2}^{+}-\mathrm{AuN}P_{\#2}^{+}$	—	—	–3,382.6 ± 11.0	–3,343.2 ± 6.1
$\mathrm{AuN}P_{\#1}^{-}-\mathrm{AuN}P_{\#1}^{-}$	–3,670.8 ± 3.9	–3,747.9 ± 4.3	–3,906.9 ± 11.0	–3,836.3 ± 7.7
$\mathrm{AuN}P_{\#1}^{-}-\mathrm{AuN}P_{\#2}^{-}$	—	—	–11.7 ± 2.7	–19.1 ± 0.8
$\mathrm{AuN}P_{\#2}^{-}-\mathrm{AuN}P_{\#2}^{-}$	—	—	–3,899.0 ± 6.0	–3,791.6 ± 5.3
$\mathrm{AuN}P_{\#1}^{+}-\mathrm{AuN}P_{\#1}^{-}$	440.3 ± 11.0	225.7 ± 5.8	351.8 ± 12.0	195.1 ± 5.0
$\mathrm{AuN}P_{\#1}^{+}-\mathrm{AuN}P_{\#2}^{-}$	—	—	–2.2 ± 7.1	124.8 ± 7.8
$\mathrm{AuN}P_{\#2}^{+}-\mathrm{AuN}P_{\#1}^{-}$	—	—	394.7 ± 11.0	419.9 ± 8.3
$\mathrm{AuN}P_{\#2}^{+}-\mathrm{AuN}P_{\#2}^{-}$	—	—	363.4 ± 6.5	–0.001 ± 0.0

a RMSDs are shown for all average interaction.

Intratype interactions between like-charged nanoparticles (i.e., AuNP^+^–AuNP^+^ and AuNP^–^–AuNP^–^) consistently exhibit strong negative *E_LJ_* across all systems, with values ranging from −3,322 to −3,909 kJ/mol, per AuNP. These results reflect substantial attractive dispersion forces, which are primarily attributed to the large surface area and high atomic density of the NPs. Notably, *E_LJ_* interactions are slightly more pronounced for negatively charged AuNPs, particularly in system 3, which involved four AuNPs in the absence of ions. This suggests that van der Waals forces contribute meaningfully to the aggregation tendencies of anionic AuNPs under low ionic strength conditions. In contrast, *E_LJ_* interactions between oppositely charged AuNPs, AuNP^+^–AuNP^–^, are consistently positive in all cases with values ranging from 226 to 554 kJ/mol, per AuNP. These values indicate net short-range repulsion due to the strong electrostatic attraction observed in the corresponding Coulomb interaction analysis (Table 2). This behavior indicates that the strong Coulombic attraction between oppositely charged nanoparticles drives them into close proximity, where the repulsive part of the van der Waals potential becomes dominant. Notably, the presence of ions in systems 2 and 4 reduces the magnitude of these repulsive *E_LJ_* contributions, likely due to ionic screening and the rearrangement of hydration shells. Interactions between AuNPs and water molecules follow an expected polarity-dependent trend consistent with expectations. Negatively charged AuNPs exhibit stronger *E_LJ_* interactions with water, with values ranging from −604 to −395 kJ/mol, per AuNP, compared to their positively charged counterparts, which range from −541 to −246 kJ/mol. These results reinforce the premise that anionic ligands (−COO^–^) are more favorable to van der Waals interactions with water molecules, likely due to the increase of HBs capacity and the formation of more structured solvation shells around nanoparticles. As anticipated, interactions between AuNPs and ions exhibit comparatively weaker *E_LJ_* contributions with values ranging from −0.3 to −25.7 kJ/mol, per AuNP. While the electrostatic forces contribution primary drives the interactions, the minor values for van der Waals attractions—most notably between AuNP*^–^* and Na^+^ (−12.6 kJ/mol in system 2) and AuNP^+^ and Cl^–^ (−25.7 kJ/mol in system 4)—indicate a secondary but complementary role of dispersion forces. These interactions likely contribute to the stabilization of local ionic environments at the NP interface.

The detailed *E_LJ_* values presented in Table 5 reveal spatial asymmetries and positional variability that are not captured by the system-wide average values. Self-interactions (e.g., $AuNP_{\#1}^{+}-AuNP_{\#1}^{+}$ ) consistently exhibit strongly negative values. These results underscore the internal robust cohesion of each AuNP. Interactions between distinct AuNPs of the same charge type (e.g., $AuNP_{\#1}^{+}-AuNP_{\#2}^{+}$ ) exhibit near-zero or weakly attractive *E_LJ_* values, ranging from −8.6 to −0.1 kJ/mol. These minimal interaction values suggest limited van der Waals contributions, likely attributable to steric separation or solvent-mediated repulsion. Notably, the *E_LJ_* values approach zero in systems containing ions, indicating enhanced spatial separation and stabilization driven by electrostatic screening and solvation effects. *E_LJ_* interactions between oppositely charged AuNPs remain predominantly positive across all configurations, indicating that short-range van der Waals repulsion outweighs any attractive dispersion forces. This behavior is likely a consequence of the structural arrangement and steric bulk of the ligand shells. Notably, specific interactions such as $AuNP_{\#2}^{+}-AuNP_{\#2}^{-}$ in system 4 yield values approaching zero (+0.001 ± 0.0004 kJ/mol), suggesting either full charge neutralization via ionic shielding or large interparticle separation within the simulation box.

Taken together, these findings underscore the secondary yet significant role of van der Waals forces in modulating the spatial organization and dynamic stability of AuNP systems in aqueous media. While Coulombic interactions govern both long-range and short-range behavior by driving charged AuNPs into close proximity, Lennard–Jones forces become significant at short distances, introducing repulsive contributions that help define steric boundaries and prevent particle overlap. The presence of ions markedly modulates both Coulombic and van der Waals interactions within the system. Electrostatic screening diminishes the attractive forces between oppositely charged particles and currently attenuates repulsive interactions among like-charged nanoparticles. Meanwhile, van der Waals forces contribute to maintaining spatial organization through excluded volume effects and structuring of the solvation shell. Collectively, these findings indicate that NP behavior in solution is governed by a delicate balance between Coulombic and dispersion interactions—an equilibrium that is crucial for predicting aggregation tendencies, ensuring colloidal stability, and optimizing functional performance in nanotechnological applications. Figure 2 demonstrates the behavior of the total potential energy (and its average) for systems 1–4 during the production process, highlighting that the systems are in a thermodynamic equilibrium state in which the above analyses were performed. We also highlight a configuration of each system, where the positioning of the AuNPs and ions in the simulation box can be observed.

**Figure 2:**
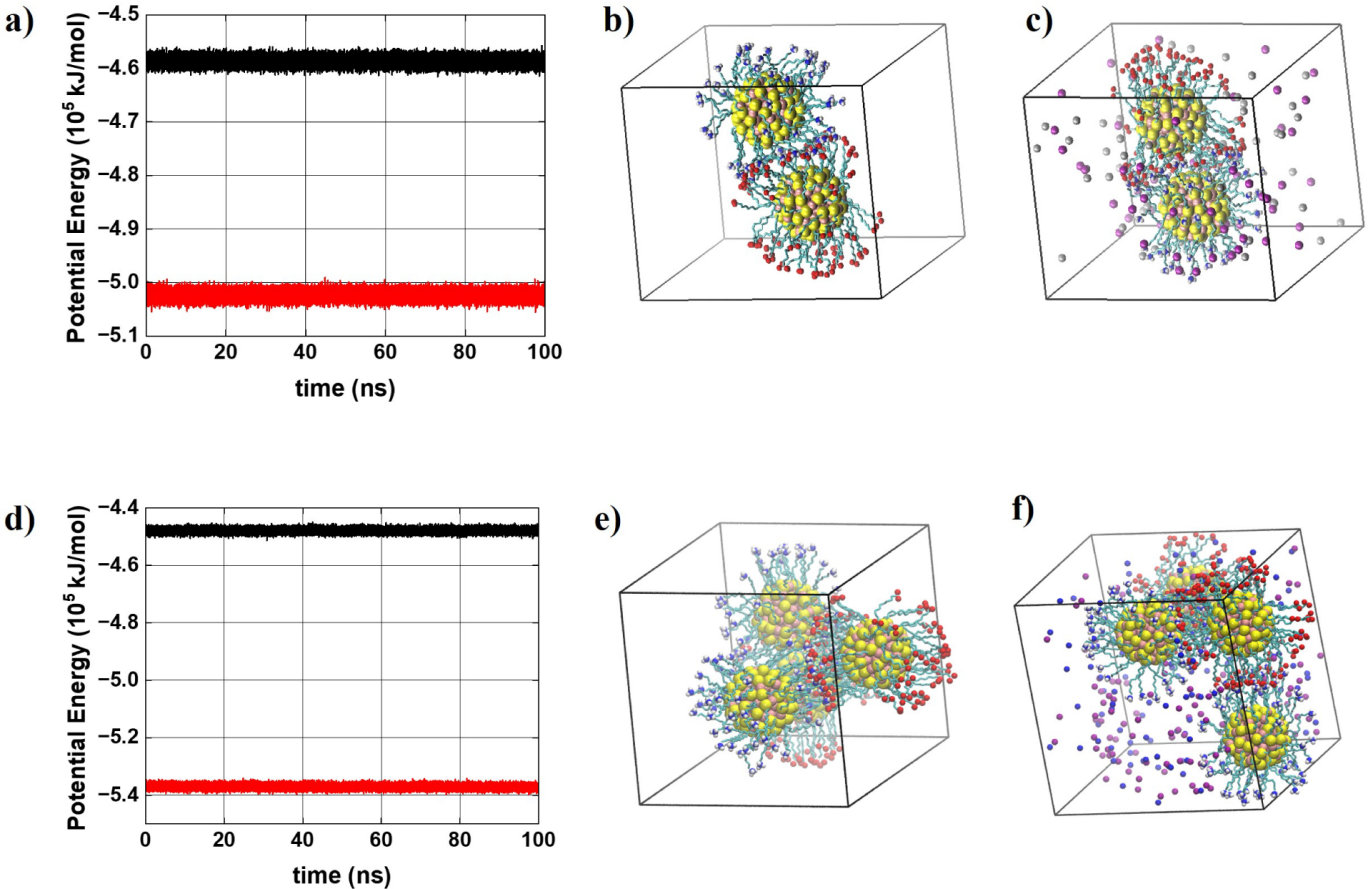
Behavior of the total potential energy for systems 1–4 during the production run (confirming that the system is in thermodynamic equilibrium during this production stage). A configuration of each system is shown, highlighting the positioning of AuNPs and ions within the simulation box. (a) Potential Energy—system 1 (black line) and system 2 (red line); (b) system 1; (c) system 2. (d) Potential energy—system 3 (black line) and system 4 (red line); (e) system 3, (f) system 4. Yellow = gold atoms; orange = sulfur atoms; cyan = carbon atoms; red = oxygen atoms; dark blue = nitrogen atoms; white = hydrogen atoms. The water molecules were intentionally omitted to enhance the visibility of the AuNPs and ions.

### 3.3 Hydrogen Bond Structure and Dynamics

HBs play a pivotal role in governing AuNP–solvent interactions, particularly in aqueous environments, where the structuring of water at the NP interface influences both dynamic behavior and interfacial stability. Table 6 presents the average number of HBs formed between functionalized AuNPs and surrounding water molecules, alongside the corresponding forward HB lifetimes and Gibbs free energy (Δ*G*) values associated with HB dissociation. These values were computed using autocorrelation function methodology as proposed by Luzar, Chandler, and van der Spoel.^72–74^ The number of HBs formed between AuNPs and water molecules varies significantly as a function of NP electric charge and system configuration. AuNPs^–^ consistently form a greater number of HBs across all systems configurations, with values ranging from 216 to 289 HBs, per AuNP, in contrast to AuNPs^+^, which form between 73 and 110 HBs with water molecules in solution. This disparity is primarily attributed to the stronger electrostatic attraction between the negatively charged surface functional groups and the partial positive charges on the hydrogen atoms of the water molecules, thereby facilitating HB formation. The inclusion of additional AuNPs (as in systems 3 and 4) or ionic species (as in systems 2 and 4) induces notable rearrangements within the HB network. For instance, in system 4, $AuNP_{\#2}^{-}$ forms the highest number of HBs (289), likely due to favorable local solvation conditions and partial shielding of neighboring particles, which facilitates increased water molecules accessibility and interaction. In contrast, positively charged AuNPs exhibit a slight reduction in HB formation in multiparticle systems, suggesting steric hindrance or ionic interference that disrupts optimal HB formation. It is important to highlight that the presence of oppositely charged AuNPs within the same simulation box leads to interactions between these particles, which reduces the average number of HBs formed with water molecules compared to a solvated environment containing only AuNPs with the same surface charge. Previous studies have shown that the HB network formed with water molecules in the vicinity of AuNPs varies between 380 and 420 HBs per AuNP,^48,49^ depending on the ionic environment and the concentration of AuNPs in solution.

**Table 6:** Average Number of Hydrogen Bonds (HBs) Formed between AuNPs and Surrounding Water Molecules along with the Corresponding Forward HB Lifetimes (in ps)^a^

#HBs				
	System 1	System 2	System 3	System 4
$\mathrm{AuN}P_{\#1}^{+}$	87.3	95.6	77.8	76.9
$\mathrm{AuN}P_{\#2}^{+}$	—	—	73.5	110.9
$\mathrm{AuN}P_{\#1}^{-}$	231.8	256.8	216.9	217.1
$\mathrm{AuN}P_{\#2}^{-}$	—	—	219.5	289.4
Lifetime [ps]				
$\mathrm{AuN}P_{\#1}^{+}$	53.0	32.0	37.5	52.9
$\mathrm{AuN}P_{\#2}^{+}$	—	—	46.0	26.8
$\mathrm{AuN}P_{\#1}^{-}$	21.3	19.7	32.1	31.1
$\mathrm{AuN}P_{\#2}^{-}$	—	—	29.1	19.6
Δ*G* [kJ/mol]				
$\mathrm{AuN}P_{\#1}^{+}$	14.4	13.1	14.0	14.4
$\mathrm{AuN}P_{\#2}^{+}$	—	—	13.8	12.7
$\mathrm{AuN}P_{\#1}^{-}$	12.1	11.9	13.1	13.0
$\mathrm{AuN}P_{\#2}^{-}$	—	—	12.9	11.9

a Average HBs were dentified based on geometric criteria: angle θ ≤ 30^°^ and interatomic distance *r* ≤ 0.35 nm. Forward HB lifetime and HB rupture Gibbs energy (Δ*G*) values for HB dissociation were calculated using autocorrelation analysis functions following the framework outlined in refs. 72–74 .

The average HB lifetimes present an inverse relationship with the number of HBs formed. AuNPs with lower HB counts, particularly those bearing positive surface charges, tend to display longer HB lifetimes. For instance, $AuNP_{\#1}^{+}$ in system 1 shows the longest observed lifetime at ∼53 ps. In contrast, negatively charged particles, despite forming a greater number of HBs, exhibit shorter HB lifetimes ranging from ∼19 to ∼32 ps, suggesting more transient interactions. This behavior reflects the influence of stronger solvation dynamics and heightened molecular flux in the vicinity of the negatively charged NP surface. Notably, the shortest HB lifetimes are observed for both $AuNP_{\#2}^{+}$ (∼26 ps) and $AuNP_{\#2}^{-}$ (∼19 ps) in system 4, suggesting that ionic screening and interparticle interactions increase HB’s dynamics. These effects accelerate the turnover of water molecules in the hydration layer, reflecting a more dynamic exchange at the NP interface.

The calculated Gibbs free energy (Δ*G*) associated with HB disruption further supports the trends observed in HB number and lifetimes. AuNP^+^ exhibit higher Δ*G* values—reaching up to ∼14 kJ/mol—indicating more stable and persistent HBs, despite their lower overall counts. In contrast, AuNP^–^ display lower Δ*G* values ranging from ∼11 to ∼13 kJ/mol, consistent with their shorter HB lifetimes and the more dynamic hydration shells surrounding these particles. These findings highlight a trade-off between the quantity and stability of HBs: while anionic AuNPs engage more extensively with water through a greater HBs, these interactions tend to be weaker and more transient. In contrast, cationic AuNPs form fewer HBs, but these are characterized by longer HB lifetimes and greater energetic stability. Overall, the analysis demonstrates that HB characteristics are strongly modulated by surface charge in combined solvation (AuNP^+^ + AuNP^–^ + *H*_2_ *O*), ionic environment, and NP–NP proximity. Variations in HB average number, HB lifetime, and Δ*G* underscore the influence of the local electrostatic environment on the structure and dynamics of the solvation shell, thereby impacting interfacial properties and colloidal stability. These findings contribute significantly to a deeper understanding of NP dispersion, reactivity, and mobility in complex aqueous environments. Moreover, they may inform the rational design of AuNP-based platforms for biomedical, catalytic, and sensing applications where solvation behavior plays a pivotal role in application.

As observed, the average number of HBs between the AuNP and water molecules is drastically reduced when compared to previous studies that considered only one type of AuNP in solution. This may indicate that the presence of AuNPs with different electrostatic natures can interact with each other, enabling the formation of HBs between AuNP pairs. In this context, our results show that the HB statistics for AuNP^+^–AuNP^–^ interactions account for approximately 95.6 HBs per pair (system 1); 82.4 HBs per pair (system 2); 104.3 HBs per pair (system 3); and 83.9 HBs per pair (system 4). This finding is particularly relevant, as it indicates that inter-NPs HBs constitute a substantial contribution to the overall structural organization of the system. Such interactions may influence key physicochemical properties, including colloidal stability, aggregation propensity, and potentially the emergent functionality of the nanomaterial. These results underscore the importance of electrostatic complementarity in modulating NP behavior and provide valuable insights for the rational design of hybrid nanostructures with tunable interfacial characteristics.

These findings also raise important considerations regarding the implications of HBs instability for the structural integrity of the solvation shell. The more transient and dynamic solvation environment observed around negatively charged AuNPs suggests a less rigid hydration layer, which may enhance the molecular exchange and surface accessibility. In biological systems, this could influence both the biocompatibility and functionalization efficiency of the NP, as a more labile solvation shell may facilitate interactions with biomolecules or ligands. However, it may also reduce the colloidal stability or alter protein corona formation in complex fluids. Therefore, understanding the balance between HB quantity and stability is crucial when tailoring the AuNP surface properties for biomedical applications.

### 3.4 Mass Density Profile

We present the mass density profile of each component comprising the systems, which reveals the structural organization of molecules and AuNPs within the simulation box. Figures 3 [Fig fig4] [Fig fig5] display the mass density distribution along X, Y, and Z, axes, respectively. To facilitate analysis, all mass density profiles are centered relative to $AuNP_{\#1}^{-}$ (black curve), which is positioned at the center of the simulation box. Figure 3 presents the mass density profiles along the *X*-axis for the components of the system, including positively and negatively charged AuNPs, ions, and water molecules. In system 1, the water density profile (blue curve) displays relatively homogeneous distribution, with noticeable depression in the central region corresponding to the location of the AuNPs. The AuNPs^–^ (black curve) and AuNPs^+^ (green curve) exhibit well-defined localized peaks (specifically for the gold atoms, primarily due to their higher atomic mass compared with the other atoms of the AuNP), indicating their central position within the system. The separation between these peaks suggests limited aggregation, which may be attributed to the absence of compensating ions. In system 2, the water density profile (blue curve) remains relatively similar to that observed in system 1, exhibiting a slight depression in the region occupied by the AuNPs. Although the mass density peaks of the AuNPs remain well defined, their profiles are slightly altered. In system 3, the mass density profiles exhibit a more complex pattern, with four distinct peaks corresponding to two AuNPs^*–*^ (black and yellow curves) and two AuNPs^+^ (green and red curves). The water density (blue curve) displays a pronounced depression near the edges of the simulation box, indicating that the AuNPs aggregation displaces surrounding solvent molecules. The proximity of AuNPs peaks suggests stronger interparticle interactions in the absence of ions likely driven by van der Waals forces and uncompensated electrostatic interactions, resulting in direct aggregation tendencies. In system 4, the water density distribution (blue curve) closely resembles that of system 3 but exhibits a slightly reduced density near the system boundaries. The AuNPs mass density peaks (black, yellow, green, and red curves) remain distinct though a subtle redistribution is observed when compared to system3. For systems 2 and 4, ion distribution reveals that Cl^–^ ions (gray curve) are concentrated in the vicinity of the AuNPs^+^, whereas Na^+^ (magenta curve) show lower overall intensity but display slightly accumulation near the AuNPs^–^. These observations suggest that electrostatic interactions between the ions and the AuNPs begin to influence the structural organization of the system.

**Figure 3:**
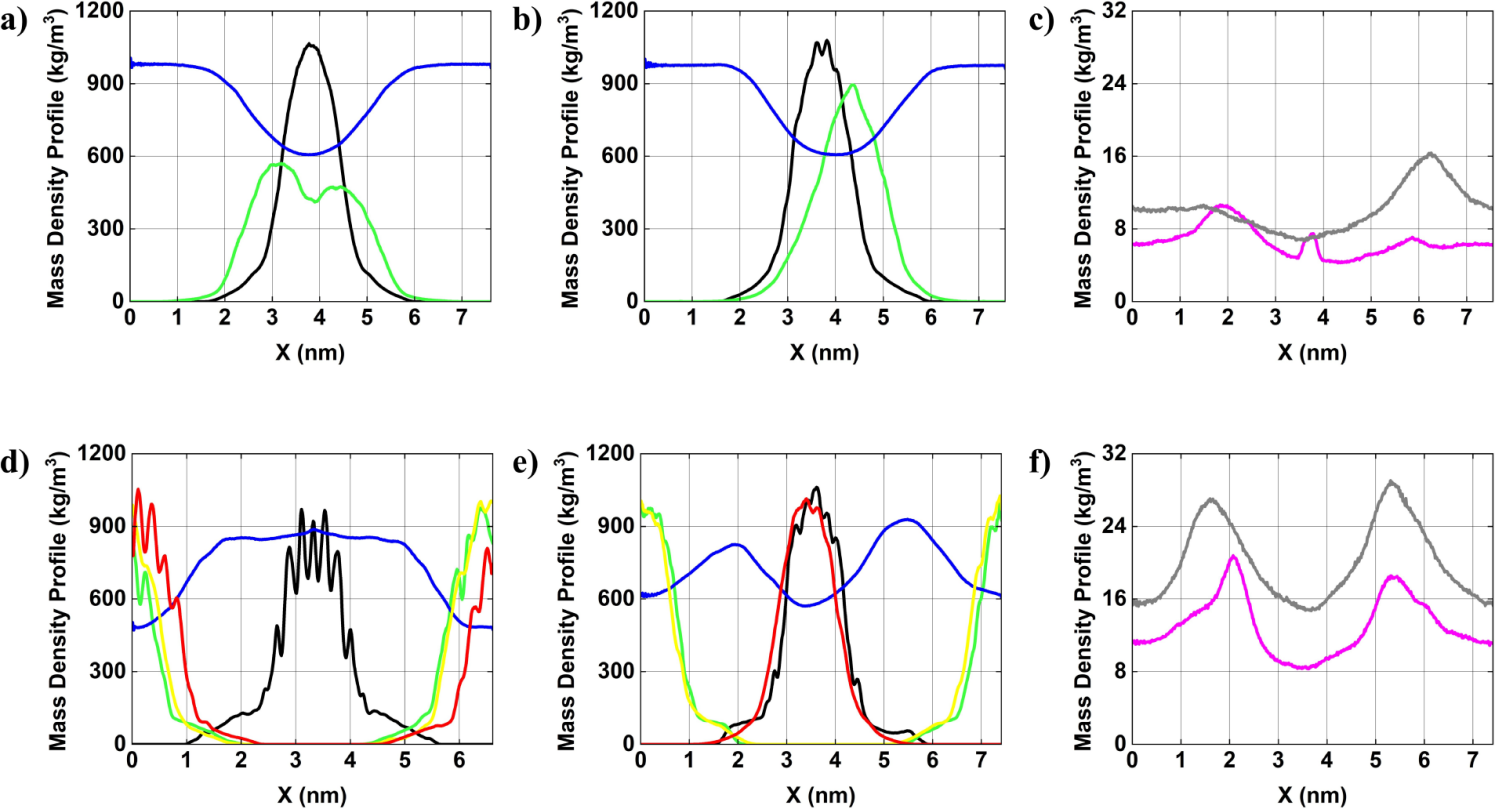
Average mass density profile [in kg/m^3^] of AuNPs and ions in solution along the *X*-axis of the simulation box. (a) system 1; (b) system 2; (c) ions for system 2; (d) system 3; (e) system 4; and (f) ions for system 4. ${\mathrm{AuNP}}_{\#1}^{-}$ = black curve; ${\mathrm{AuNP}}_{\#1}^{+}$ = green curve; ${\mathrm{AuNP}}_{\#2}^{-}$ = yellow curve; ${\mathrm{AuNP}}_{\#2}^{+}$ = red curve; water = blue curve; ion Cl^–^= gray curve; and ion Na^+^ = magenta curve.

**Figure 4:**
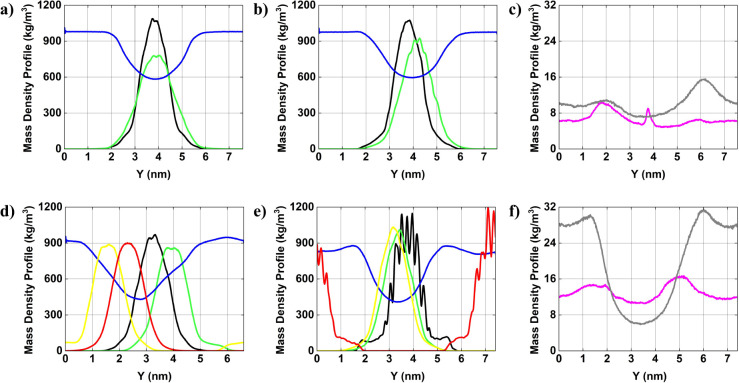
Average mass density profile [in kg/m^3^] of AuNPs and ions in solution along the *Y*-axis of the simulation box. (a) system 1; (b) system 2; (c) ions for system 2; (d) system 3; (e) system 4; and (f) ions for system 4. ${\mathrm{AuNP}}_{\#1}^{-}$ = black curve; ${\mathrm{AuNP}}_{\#1}^{+}$ = green curve; ${\mathrm{AuNP}}_{\#2}^{-}$ = yellow curve; ${\mathrm{AuNP}}_{\#2}^{+}$ = red curve; water = blue curve; ion Cl^–^= gray curve; and ion Na^+^ = magenta curve.

**Figure 5:**
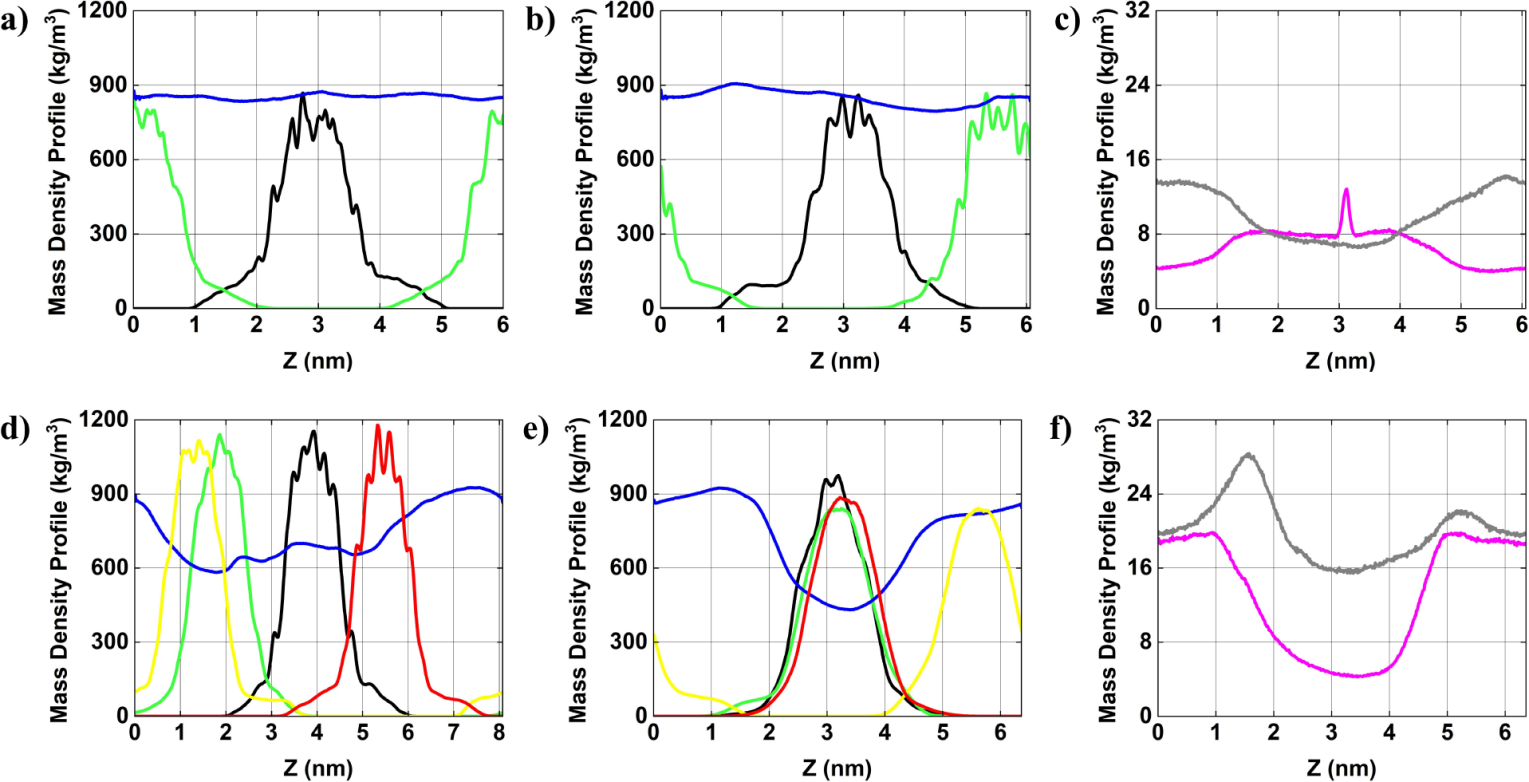
Average mass density profile [in kg/m^3^] of AuNPs and ions in solution along the *Z*-axis of the simulation box. (a) system 1; (b) system 2; (c) ions for system 2; (d) system 3; (e) system 4; and (f) ions for system 4. ${\mathrm{AuNP}}_{\#1}^{-}$ = black curve; ${\mathrm{AuNP}}_{\#1}^{+}$ = green curve; ${\mathrm{AuNP}}_{\#2}^{-}$ = yellow curve; ${\mathrm{AuNP}}_{\#2}^{+}$ = red curve; water = blue curve; ion Cl^–^= gray curve; and ion Na^+^ = magenta curve.

Figure 4 displays the mass density distribution of AuNPs and water molecules along the *Y*-axis. In system 1, the density profiles of the AuNPs^–^ (black curve) and the AuNPs^+^ (green curve) exhibit well-defined peaks, indicating that both AuNPs are localized in approximately the same region along *y*-axis. The water density profile (blue curve) shows a slight depression in the central region, which suggests a displacement of water molecules due to the presence of AuNPs. In system 2, the mass density distribution exhibits a pattern comparable to that observed in system 1. The density peaks corresponding to the AuNPs (black and green curves) remain well-defined, indicating no significant deviations in the spatial arrangement of the AuNPs. The water density profile exhibits consistent behavior, with a slight depression in the central region, indicative of partial solvent exclusion in the vicinity of the AuNPs. In the accompanying graph, the ion profiles reveal that Cl^–^ ions (gray curve) are concentrated near the AuNPs^+^, whereas the Na^+^ ions (magenta curve) although present at lower concentrations, show a modest accumulation near the AuNPs^–^. The introduction of ions can induce electrostatic interactions that subtly affect the spatial organization of the AuNPs and the surrounding solvent; however, these interactions do not lead to significant structural alterations within the system. In system 3, the mass density distribution graph reveals four distinct peaks corresponding to $AuNPs_{\#1}^{-}$ (black curve), $AuNPs_{\#2}^{-}$ (yellow curve), $AuNPs_{\#1}^{+}$ (green curve), and $AuNPs_{\#2}^{+}$ (red curve). The AuNPs are distributed along *y*-axis with minimal overlap between their respective mass density profiles, indicating that the AuNPs remain well separated in the simulation box. The water density profile (blue curve) exhibits a more pronounced central depression, likely indicating increased solvent displacement due to the higher concentrations of AuNPs. In system 4, the four distinct density peaks corresponding to the AuNPs remain observable; however, slight peak variations relative to Figure 3d suggest potential *NP* reorganization induced by the presence of ions. The water profile (blue curve) maintains a similar trend comparable to that observed in system 3, with a persistent central density reduction. In the accompanying graph, the ion profiles reveal that Cl^–^ ions (gray curve) exhibit stronger accumulation near AuNPs^+^, indicative of a stabilizing electrostatic interaction. Sodium ions Na^+^ (magenta curve) display more homogeneous distribution overall, though with slight enrichment near the AuNPs^–^.

Figure 5 presents the mass density distribution profiles of AuNPs and water along the *Z*-axis across four system configurations. In system 1, the AuNPs^–^ (black curve) and the AuNPs^+^ (green curve) exhibit well-defined density peaks, indicating that the particles are spatially separated along *Z*-axis. The water density profile (blue curve) is relatively homogeneous, suggesting a minimal solvent density profile perturbation in this direction. This spatial separation implies moderated interaction between the NP under these conditions. In system 2, the density peaks of the AuNPs remain well defined and occupy positions similar to those in system 1, indicating stability in their spatial arrangement. The water density profile is also consistent with the previous configuration. In the corresponding ion density graph, Cl^–^ ions (gray curve) exhibit pronounced accumulation near the AuNPs^+^ while Na^+^ ions (magenta curve), though present at lower concentration, exhibit modest accumulation near the AuNPs^–^. In system 3, four distinct peaks are observed, corresponding to the presence of multiple AuNPs. Although the AuNPs are distributed along *Z*-axis, slight overlaps in their density profiles suggest localized interactions between some particles. The water density profile (blue curve) features multiple depressions, indicating substantial displacement of solvent molecules in regions occupied by the AuNPs. The increased number of AuNPs leads to a more complex spatial distribution and enhances the possibility of interactions between oppositely charged particles. In system 4, the four peaks remain discernible but exhibit slight shifts in both position and intensity, suggesting that the ion presence influences the spatial organization of the AuNPs. The water density profile appears less irregular compared to system 3, implying that the ions may mitigate solvent exclusion in the central region of the simulation box. In the lower panel, Cl^–^ ions (gray curve) accumulate strongly around AuNPs^+^ and Na^+^ ions (magenta curve) display a similar, but less intense accumulation around AuNPs^–^. Overall, AuNPs exhibit well-defined spatial distributions along *z*-axis, appearing more ordered in the absence of ions and slightly redistributed when ions are presented. Water density consistently decreases in regions occupied by the AuNPs, particularly in systems with higher AuNPs concentrations. The presence of Cl^–^ and Na^+^ ions modulate AuNPs distribution by reducing direct aggregation and promoting electrostatic stabilization.

The results indicate that the peaks observed in the mass density profiles enable the estimation of the diameter and volume occupied by the center core of the AuNPs. This estimation is based on the full width at half-maximum (fwhm) of the curves projected along each spatial axis, as summarized in Table 7. The fwhm is defined as the distance between two points in a curve where the mass density reaches half of its maximum value. It is worth noting that the gold core, due to its higher atomic mass, contributes more significantly to the mass density profile than the organic functionalization shell, which explains the sharper and more pronounced central peak observed in the distributions. The calculated average diameter (*d̅*) ranges from 1.30 and 1.52 nm, corresponding to estimated average volumes (*V̅*) between 1.15 nm^3^ and 1.84 nm^3^. Among the analyzed configurations, system 1 exhibits the highest fwhm value of *d̅* and *V̅*, suggesting that the AuNPs in this system may be more expanded or undergoing distinct interactions. In contrast, system 3 and system 4 display the lowest values, indicative of a more compact NP structure. The observed variations in *d̅* and *V̅*values across different configurations point to structural changes in the AuNPs, which appear to be influenced by the specific characteristics of each system. These differences may arise from factors such as AuNPs aggregation, electrostatic interactions with surrounding ions, or internal structural rearrangement of AuNPs within the solvent medium. A visual inspection of the simulated configurations offers additional insight into the spatial organization of the AuNPs and their interaction with ions, supporting the interpretation of the computational results. To further illustrate these findings, Figure 6 presents some representative MD snapshots extracted from classical trajectories, with the AuNPs visually highlighted. In addition, an analysis of interparticle distances provides quantitative information on the spatial correlation and interaction patterns among AuNPs. These results are in agreement with experimental findings, with core sizes of 1.6 ± 0.3.^76^

**Table 7:** Average Values for the Diameter (*d̅*) and Volume (*V̅*) of AuNPs Calculated from the Mean FWHM Values and the Mass Density Profiles along the X, Y, and *Z*-Axes of Simulation Box^a^

FWHM				
	System 1	System 2	System 3	System 4
$\mathrm{AuNP}s_{\#1}^{+}$	1.73	1.51	1.31	1.34
$\mathrm{AuNP}s_{\#2}^{+}$	—	—	1.31	1.30
$\mathrm{AuNP}s_{\#1}^{-}$	1.31	1.33	1.26	1.22
$\mathrm{AuNP}s_{\#2}^{-}$	—	—	1.33	1.34
*d̅* (in nm)	1.52	1.42	1.30	1.30
*V̅* (in nm^3^)	1.84	1.50	1.15	1.15

a The volume was estimated by assuming a spherical geometry for each AuNP.

**Figure 6:**
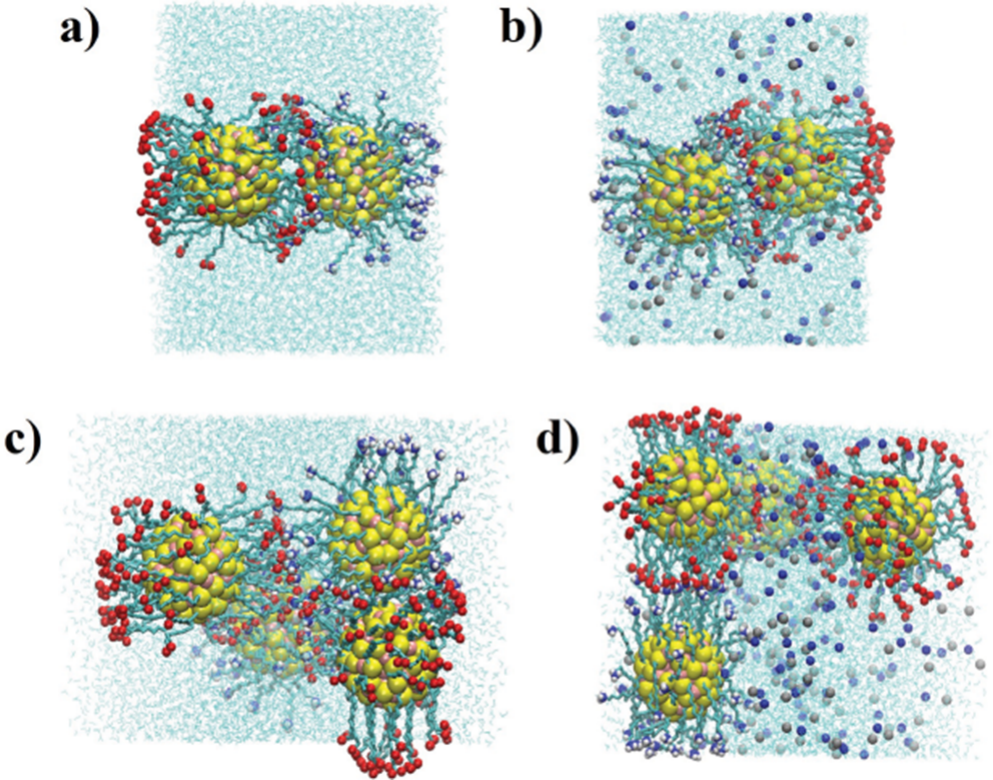
Structural details of the AuNPs–ion cluster configurations. Each AuNP is represented by the molecular formula ${\mathrm{Au}}_{144}{\left({\mathrm{SRNH}}_{3}^{+}\right)}_{60}$ where ligand consists of an alkyl chain *R* =*C*_11_ *R*_22_ (green) terminating in a polar group ( $-{\mathrm{NH}}_{3}^{+}$ or −COO^–^). The ligands are connected to the gold core *Au*_144_ (yellow) via sulfur atoms S (orange). The panels illustrate the molecular organization of the system in different configurations: (a) system 1, (b) system 2, (c) system 3, and (d) system 4. Yellow = gold atoms; orange = sulfur atoms; cyan = carbon atoms; red = oxygen atoms; dark blue = nitrogen atoms; white = hydrogen atoms. Water molecules are represented by blue lines.

### 3.5 Distance between AuNPs

Figures 7–9 depict the time evolution of distances between the centers of mass of selected atom groups obtained from MD simulation. Figure 7 specifically presents interparticle distances between the $AuNPs_{\#1}^{-}$ and the $AuNPs_{\#1}^{+}$ (black line), across all four configurations. The corresponding average distances are indicated in red: (a) ∼3.0 nm, (b) ∼2.6 nm, (c) ∼3.8 nm, and (d) ∼3.4 nm. A comparison between system 1 and system 2 reveals a slight decrease in the average distance between the oppositely charged AuNPs. This reduction may be attributed to the introduction of ions at low concentration, which potentially leads to an approximation of the oppositely charged AuNPs due to partial shielding of the charges. In system 3, which includes higher molar concentration of AuNPs, the average distance between $AuNPs_{\#1}^{-}$ and $AuNPs_{\#1}^{+}$ is the largest among all configurations (∼3.8 nm). This increase may result from the influence of additional AuNPs, which can disturb the local electrostatic field, thus preventing close interaction of the specific pair under analysis. In system 4, characterized by both higher AuNP and ion concentration, the intermediate average distance is approximately ∼3.4 nm. This value exceeds that observed in system 2 (with lower ion concentration) but is less than that of system 3 (no ions and higher molar concentration of AuNPs). The observed trend suggests that increased ion concentration enhances electrostatic screening weakening the attractive interactions between oppositely charged AuNPs and thus increasing their average distance relatively to system 2. Overall, these observations suggest that the presence of only a single pair of oppositely charged AuNPs (systems-01 and 02) tend to support shorter interparticle distances. Conversely, systems with higher particle concentrations (systems-03 and 04) display more complex behavior.

**Figure 7:**
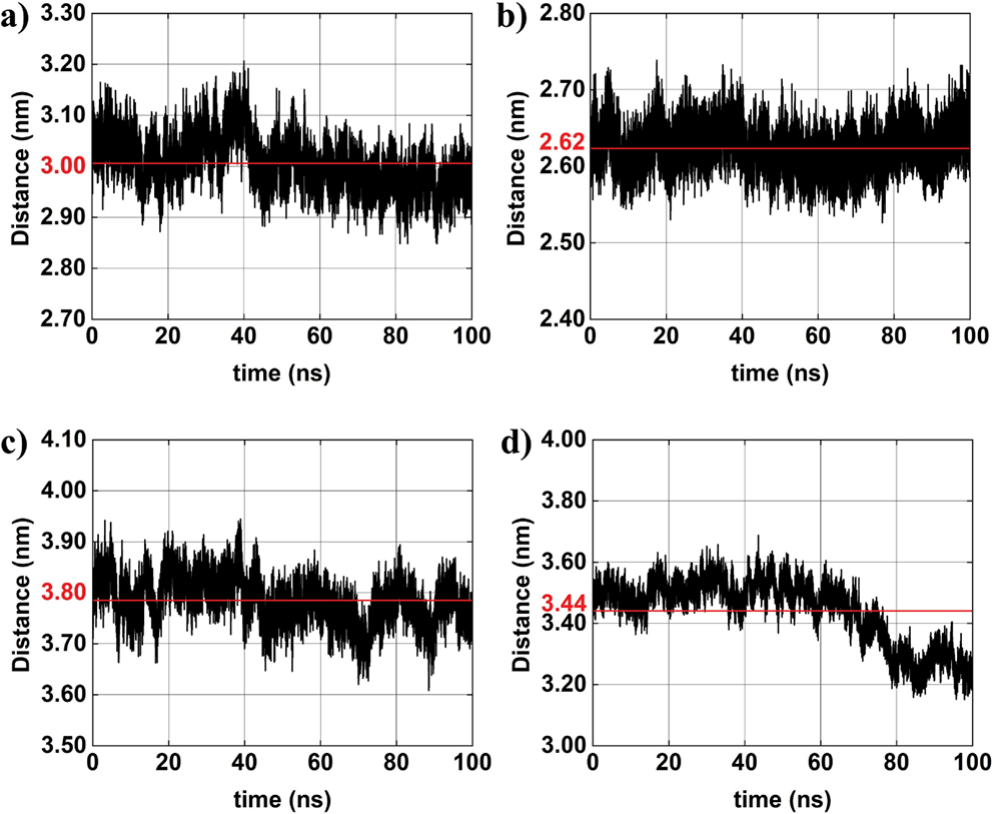
Interparticle distances between charged AuNPs $\left({\mathrm{AuNP}}_{\#1}^{-}-{\mathrm{AuNP}}_{\#1}^{+}\right)$ obtained from *MD* simulations (black lines): (a) system 01; (b) system 02; (c) system 03; and (d) system 04. The red horizontal line in each panel represents the average distance over the simulation time.

**Figure 8:**
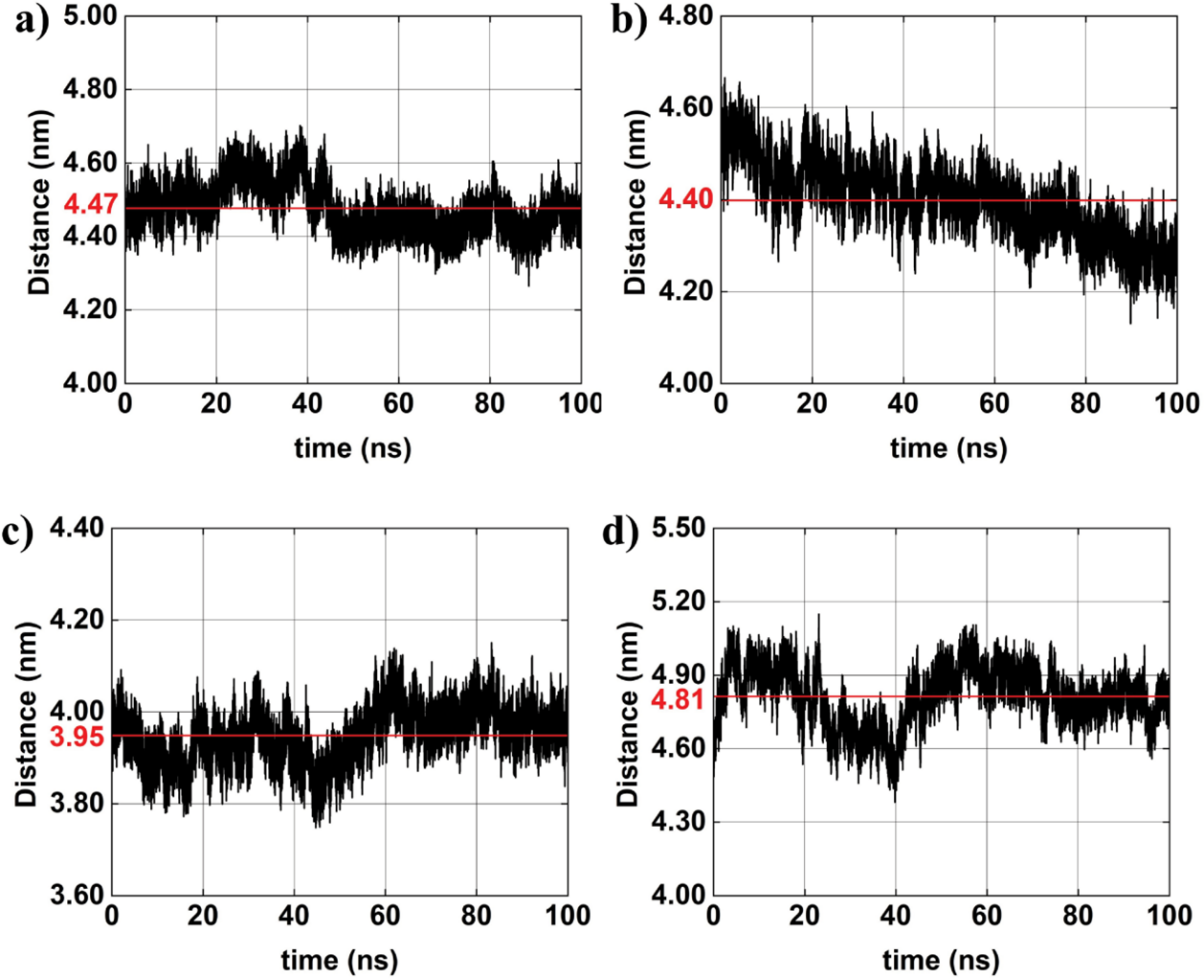
AuNPs *o*btained from *MD* simulations (black lines): (a) system 3: $AuNP_{\#1}^{-}-AuNP_{\#2}^{-}$ ; (b) system 4: $AuNP_{\#1}^{-}-AuNP_{\#2}^{-}$ ; (c) system 3: $AuNP_{\#1}^{+}-AuNP_{\#2}^{+}$ ; and (d) system 4: $AuNP_{\#1}^{+}-AuNP_{\#2}^{+}$ (black). The red horizontal line in each panel represents the average distance over the simulation time.

**Figure 9:**
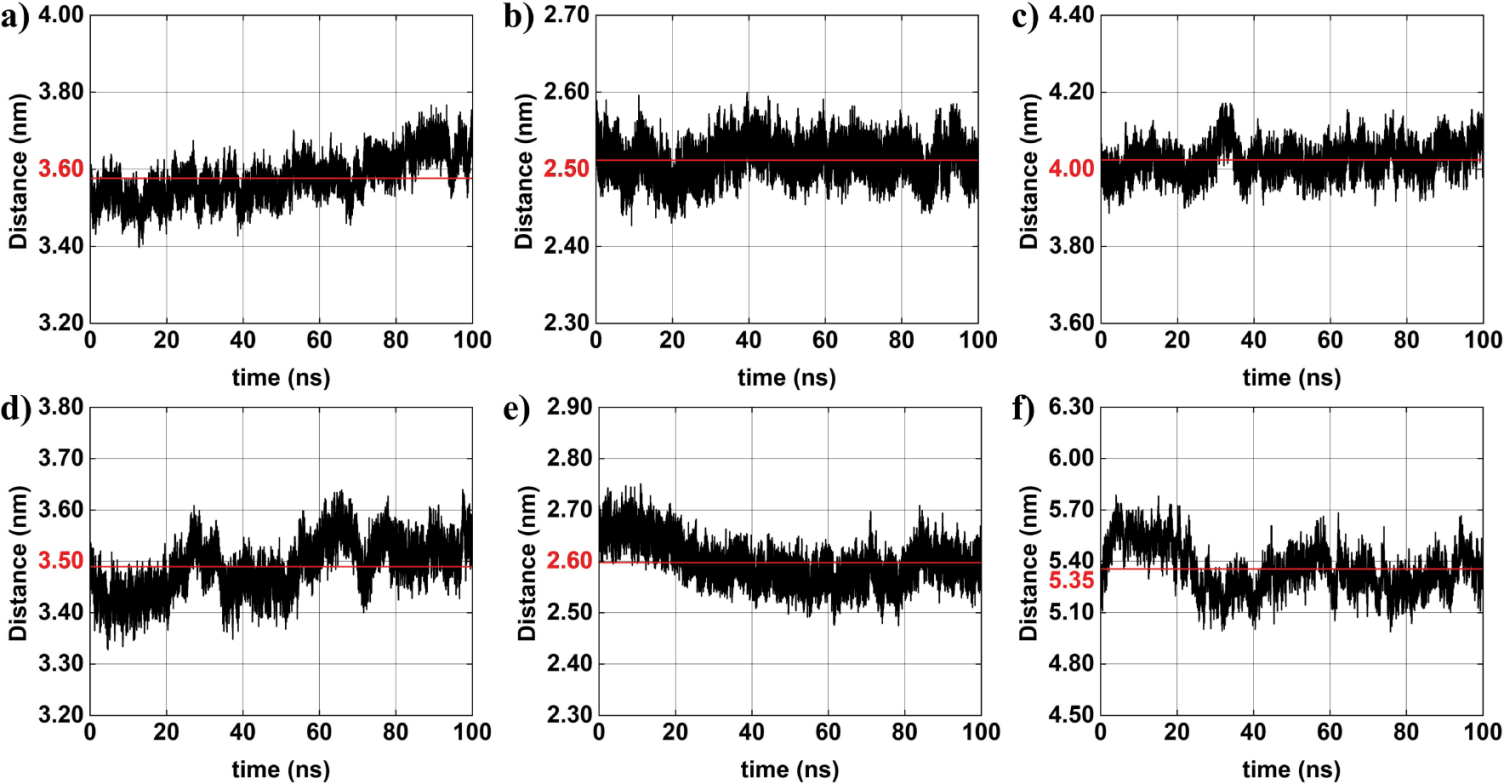
Interparticle distances between charged AuNPs obtained from *MD* simulations (black lines): (a) system 3: ${\mathrm{AuNP}}_{\#1}^{-}-{\mathrm{AuNP}}_{\#2}^{+}$ ; (b) system 3: ${\mathrm{AuNP}}_{\#2}^{-}-{\mathrm{AuNP}}_{\#1}^{+}$ ; (c) system 3: ${\mathrm{AuNP}}_{\#2}^{-}-{\mathrm{AuNP}}_{\#2}^{+}$ ; (d) system 4: ${\mathrm{AuNP}}_{\#1}^{-}-{\mathrm{AuNP}}_{\#2}^{+}$ ; (e) system 4: ${\mathrm{AuNP}}_{\#2}^{-}-{\mathrm{AuNP}}_{\#1}^{+}$ ; and (f) system 4: ${\mathrm{AuNP}}_{\#2}^{-}-{\mathrm{AuNP}}_{\#2}^{+}$ . The red horizontal line in each panel represents the average distance over the simulation time.

Figure 8 displays the time-dependent distances between the centers of mass of like-charged AuNPs pairs—namely, $AuNP_{\#1}^{-}-AuNP_{\#2}^{-}$ and $AuNP_{\#1}^{+}-AuNP_{\#2}^{+}$ —for systems 3 and 04. The corresponding average values are indicated in red: (a) ∼4.5 nm, (b) ∼4.4 nm, (c) ∼3.9 nm, and (d) ∼̃4.8 nm. In system 3, the average distance between the two AuNP*^–^* (AuNP^+^ *)* is approximately 4.5 nm (3.9 nm). These values reflect the expected electrostatic repulsion between like-charged AuNP, with the slightly greater separation observed between AuNP^–^. In system 4, which features a higher ionic concentration, the average distance between the two AuNP^–^ (AuNP^+^ *)* is approximately 4.4 nm (4.8 nm). Compared to system 3, the presence of a greater ion concentration appears to induce a modest increase in the separation between AuNP^–^ and, more notably, between the AuNP^+^. Figure 9 presents the time-dependent distances between AuNPs in systems 3 and 4: $AuNP_{\#1}^{-}-AuNP_{\#2}^{+}$ , $AuNP_{\#2}^{-}-AuNP_{\#1}^{+}$ , and $AuNP_{\#2}^{-}-AuNP_{\#2}^{+}$ . The corresponding average distances are indicated in red for system 3: (a) ∼3.6 nm, (b) ∼2.5 nm, and (c) ∼4.0 nm; for system 4: (d) ∼3.5 nm, (e) ∼2.6 nm, and (f) ∼5.4 nm. These results demonstrate that the presence of ions can either increase or decrease interparticle distances, depending on the specific charge distribution of the AuNPs, and the ionic strength of the surrounding environment. Ion-mediated screening may attenuate electrostatic repulsion between similarly charged particles or weaken attractive interactions between oppositely charged pairs, resulting in variations in the interparticle spacing. In multi-NPs systems such as systems 3 and 4, the interactions are inherently more complex. The distinct average distances observed across different pairs reflect the influence of geometric arrangement and neighboring interactions on the overall behavior of the system (Table 8). Notably, system 4, which features a higher ion concentration, demonstrates a more pronounced effect on interparticle separation. This is particularly evident in the case of the $AuNP_{\#2}^{-}-AuNP_{\#2}^{+}$ pair, where the average distance increases significantly.

**Table 8:** Averaged Interparticle Distances Derived from the Positions of the First Peak in the Radial Distribution Function (RDF), Representing the Estimated Separation between AuNPs in Systems 1–4

	System 1	System 2	System 3	System 4
$\mathrm{AuN}P_{\#1}^{-}-\mathrm{AuN}P_{\#1}^{+}$	3.10	2.88	3.88	3.58
$\mathrm{AuN}P_{\#1}^{-}-\mathrm{AuN}P_{\#2}^{+}$	—	—	3.60	3.49
$\mathrm{AuN}P_{\#1}^{-}-\mathrm{AuN}P_{\#2}^{-}$	—	—	4.52	4.63
$\mathrm{AuN}P_{\#2}^{-}-\mathrm{AuN}P_{\#1}^{+}$	—	—	2.56	2.68
$\mathrm{AuN}P_{\#2}^{-}-\mathrm{AuN}P_{\#2}^{+}$	—	—	3.96	5.40
$\mathrm{AuN}P_{\#1}^{+}-\mathrm{AuN}P_{\#2}^{+}$	—	—	4.03	4.60

### 3.6 Mean Square Displacement

In this section, we examine the Einstein diffusion coefficient (Figure 10), calculated via the mean square displacement (MSD) method (see Table 9), to assess the mobility of AuNP and ions across the four simulated systems. In system 1, the MSD profile indicates relatively high mobility for both AuNP^–^ (black curve) and AuNP^+^ (green curve). A difference in diffusion behavior is observed with a percentage variation of approximately ∼32%. In system 2, the introduction of ions results in a decrease in AuNP mobility. This reduction occurs with an ∼21% difference in diffusion coefficients between the two AuNP. The diminished variations indicate a more balanced mobility under moderate ionic concentration. In system 3, which includes a higher concentration of AuNP*s*, a pronounced reduction in NP mobility is observed. The MSD curves show significantly constrained motion, indicative of strong interparticle interactions. Compared with system 1, this corresponds to a reduction of approximately ∼91% in average AuNP mobility. In system 4, where both ions and AuNPs concentration are elevated, mobility is further diminished. This configuration yields the lowest diffusion coefficients for both species: a reduction of ∼92% for AuNPs and ∼90% for ions compared to system 2. These results highlight the role of ionic strength in limiting both AuNPs and ion mobility. Overall, increasing the molar concentration of AuNP substantially reduces their mobility, due to enhanced interparticle interactions. The addition of ions further restricts mobility, particularly system 4, where ion-induced screening stabilizes AuNPs and reduces their displacement. Among the ions, Cl^–^ consistently exhibits higher mobility than Na^+^, though both show reduced diffusion with increasing ionic strength. The marked low mobility observed in system 4 confirms that high ion concentration stabilizes the system by suppressing translational dynamics. Additionally, the interaction ions with the AuNP surface—mediated by Coulombic and van der Waals forces—contributes significantly to the observed trend of mobility. These findings align with previous results, e.g., ref.^48^, which reported reduced mobility of AuNP^–^ in Na^+^-rich aqueous environments as particle and ion concentrations increase.

**Figure 10:**
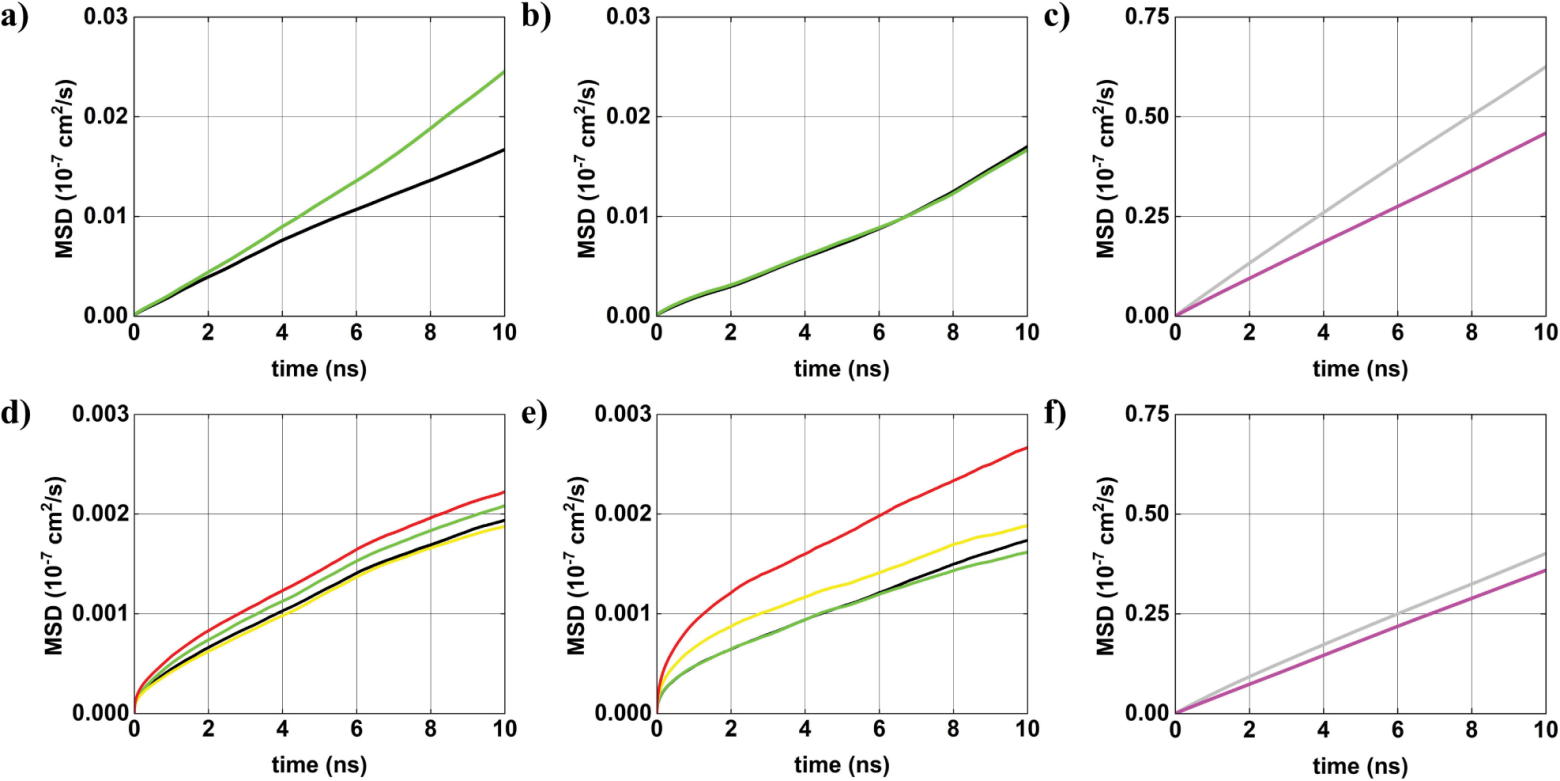
MSD curves obtained from MD simulations for AuNPs and ions: (a) system 1 for AuNPs, (b) system 2 for AuNPs, (c) system 2 for ions, (d) system 3 for AuNPs, (e) system 4 for AuNPs, and (f) system 4 for ions. ${\mathrm{AuNP}}_{\#1}^{-}$ = black curve; ${\mathrm{AuNP}}_{\#1}^{+}$ = green curve; ${\mathrm{AuNP}}_{\#2}^{-}$ = yellow curve; ${\mathrm{AuNP}}_{\#2}^{+}$ = red curve; ion Cl^–^= gray curve; and ion Na^+^ = magenta curve.

**Table 9:** Einstein Diffusion Coefficient for Each NP and Ionic Species in Systems 1–4, in Aqueous Solution^a^

	System 1	System 2	System 3	System 4
Ions Cl^–^	—	155.22 ± 9.73	—	98.05 ± 9.76
Ions Na^+^	—	113.45 ± 0.13	—	89.76 ± 2.56
Average	—	134.33 ± 4.93	—	93.90 ± 6.16
$\mathrm{AuN}P_{\#1}^{-}$	4.08 ± 0.89	4.03 ± 1.46	0.44 ± 0.15	0.37 ± 0.08
$\mathrm{AuN}P_{\#1}^{+}$	6.04 ± 1.11	3.92 ± 1.16	0.47 ± 0.18	0.34 ± 0.14
$\mathrm{AuN}P_{\#2}^{-}$	—	—	0.43 ± 0.15	0.36 ± 0.16
$\mathrm{AuN}P_{\#2}^{+}$	—	—	0.49 ± 0.19	0.51 ± 0.21
Average	5.06 ± 1.00	3.97 ± 1.31	0.46 ± 0.17	0.39 ± 0.15

a Values are reported in units of 10^–7^ cm^2^/s and refer to both AuNP and ions (Na^+^and Cl^–^).

The consistently higher diffusion coefficients observed for Cl^–^ ions compared to Na^+^ across ionic systems may be understood in light of their distinct interactions with the AuNP surfaces (see Table 9). The diffusion coefficients of Na^+^ and Cl^–^ are consistent with the values reported by Heikkilä et al.^47^ for system 2, where the increase in concentration does not significantly alter their mobility. However, in system 4, these values decrease. This reduction can be attributed to the higher density of AuNPs and counterions, which enhances electrostatic interactions and leads to more confined ionic environments near the nanoparticle surfaces. The stronger ion–AuNP association, combined with increased ion–ion correlations, results in more localized solvation structures and reduces the translational freedom of the ions. Additionally, the closer spatial arrangement of AuNPs at higher concentrations may promote confinement effects, further restricting ionic mobility. These combined factors explain the lower diffusion coefficients observed in system 4. As indicated by the density profiles, Na^+^ ions exhibit stronger electrostatic affinity toward negatively charged AuNP^–^, forming more localized and stable coordination environments that constrain their translational motion. In contrast, Cl^–^ ions, although attracted to AuNPs^+^, tend to remain more spatially diffuse, likely due to less restrictive hydration and weaker confinement at the NP interface. These differences in electrostatic proximity and solvation dynamics contribute to the enhanced mobility of Cl^–^ relative to Na^+^, despite their similar ionic radii and valence. The decrease in the Einstein diffusion coefficients with increasing ionic concentration can be attributed to the stronger electrostatic interactions and confinement effects that emerge in more concentrated systems, consistent with the results of G. Bordoni and G. Colherinhas.^48^

## 4 Conclusions

In this study, we employed *MD* simulations to investigate the energetic, structural, and dynamic between functionalized $Au_{144}{\left(SRNH_{3}^{+}\right)}_{60}$ and Au_144_(SRCOO^–^)_60_, in aqueous solution, containing varying concentrations of Na^+^ *and* Cl^–^ ions. Energetic analysis revealed that electrostatic forces play a dominant role in the long-range attraction between oppositely charged AuNPs. However, short-range stabilization is significantly influenced by steric hindrance and ligand-shell organization, which can counter van der Waals attractions and limit direct contact. Therefore, both electrostatics and dispersion forces contribute in a complementary manner to the structural arrangement and stability of the system, depending on the interparticle distance and ionic strength. The introduction of ions partially screened these electrostatic attractions, thereby reducing their strength. Additionally, increased NP concentrations led to competition for hydration by water molecules, resulting in diminished solvation energy. Ion–AuNP interactions intensified with rising AuNP concentration, indicating the adsorption of ions onto the AuNP surface. While Lennard–Jones interaction energies followed similar trends, they were of significantly lower magnitude, underscoring the predominance of electrostatic contributions in system stabilization. These findings contribute to the broader comprehension of colloidal stability and molecular-level interactions in biologically relevant aqueous environments, which is essential for optimizing applications in nanomedicine, biosensing, catalysis, and targeted drug delivery systems.

HB analyses showed that AuNP^–^ formed a greater number of HBs with water molecules compared to positively charged ones. However, these bonds were less stable, with shorter HB lifetimes and lower Gibbs free energy (Δ*G*). The presence of ions increased the total number of HBs but concurrently reduced their stability. At higher AuNP concentrations, the influence of ions was diminished due to compensatory interactions arising from the denser AuNP network. Notably, HBs were more persistent in ion-free systems, although ions contributed to localized stabilization under higher-density conditions. These results are relevant to understanding how the charge of AuNP and the presence of ions influence the structure of water around the AuNP, impacting potential biomedical and nanotechnological applications.

Spatial distribution analyses, based on mass density profiles, enable the estimation of AuNP size—yielding diameters between ∼1.3 and ∼ 1.5 nm and corresponding volumes from ∼1.1 nm^3^ to ∼1.8 nm^3^. System 2 exhibits the largest particle dimensions, likely due to structural expansion or enhanced interactions, whereas system 3 presents more compact NP arrangements. Interparticle distances varied as a function of the charge, ion concentration, and particle number, ranging from 2.5 to 5.4 nm. Notably, higher ionic concentrations—especially in system 4—resulted in greater charge screening, which modulated electrostatic repulsion and attraction, thus affecting NP organization. Mobility analyses using Einstein diffusion coefficients derived from mean square displacement (MSD) calculations confirm a reduction in AuNP mobility with increasing NP and ion interaction. These findings align with previous results,^48^ which reported reduced mobility of negatively charged AuNP in Na^+^-containing aqueous environments. In our simulations, Cl^–^ ions were consistently more mobile than Na^+^ ions, although both exhibited decreased diffusion at elevated ionic strengths. These behaviors highlight the significant role of surface-specific interactions, including electrostatics and van der Waals forces, in determining the particle and ion mobility.

Collectively, this study offers valuable insights into the complex interplay between charge, ion content, and concentration in AuNP systems. These findings have meaningful implications for the design and optimization of AuNP-based materials in nanomedicine, biosensing, catalysis, and controlled drug delivery. Importantly, these results underscore the need to consider ionic strength as a critical design parameter in the development of AuNP-based platforms intended for real-world applications. In physiological environments, where salt concentrations are inherently high, the observed reduction in NP mobility and increased tendency toward aggregation may compromise the performance of AuNPs as drug carriers or biosensors. Thus, strategies that preserve colloidal stability—such as optimizing surface functionalization to enhance steric or electrostatic repulsion—are essential to ensure effective function under biologically relevant conditions. This study provides a molecular-level perspective that can guide the rational design of robust nanomaterials for reliable operation in complex ionic environments.

While our simulations provide mechanistic insights into AuNP behavior, they are inherently constrained by the use of fixed-charge, nonpolarizable force fields and by the accessible simulation time scales. These limitations may affect the quantitative accuracy of the interaction energies and the long-term dynamics of aggregation. Future studies could incorporate polarizable force fields, reactive models, or enhanced sampling techniques to capture polarization effects, rare events, and longer-timescale processes. Additionally, exploring the influence of more complex biological environments—such as multivalent ions, mixed electrolytes, or biomolecular corona formation—would further extend the relevance of these findings to real-world nanomedicine and biosensing applications.
